# Supplementary material for: Proanthocyanidins Modulate Rumen Enzyme Activities and Protein Utilization In Vitro
Source: Molecules. 2022 Sep 10;27(18):5870. doi: 10.3390/molecules27185870 (PMC9505871; doi:10.3390/molecules27185870)
Supplement: Supplementary file 1 [file molecules-27-05870-s001.zip › Supplementary Figure_PK.pdf]

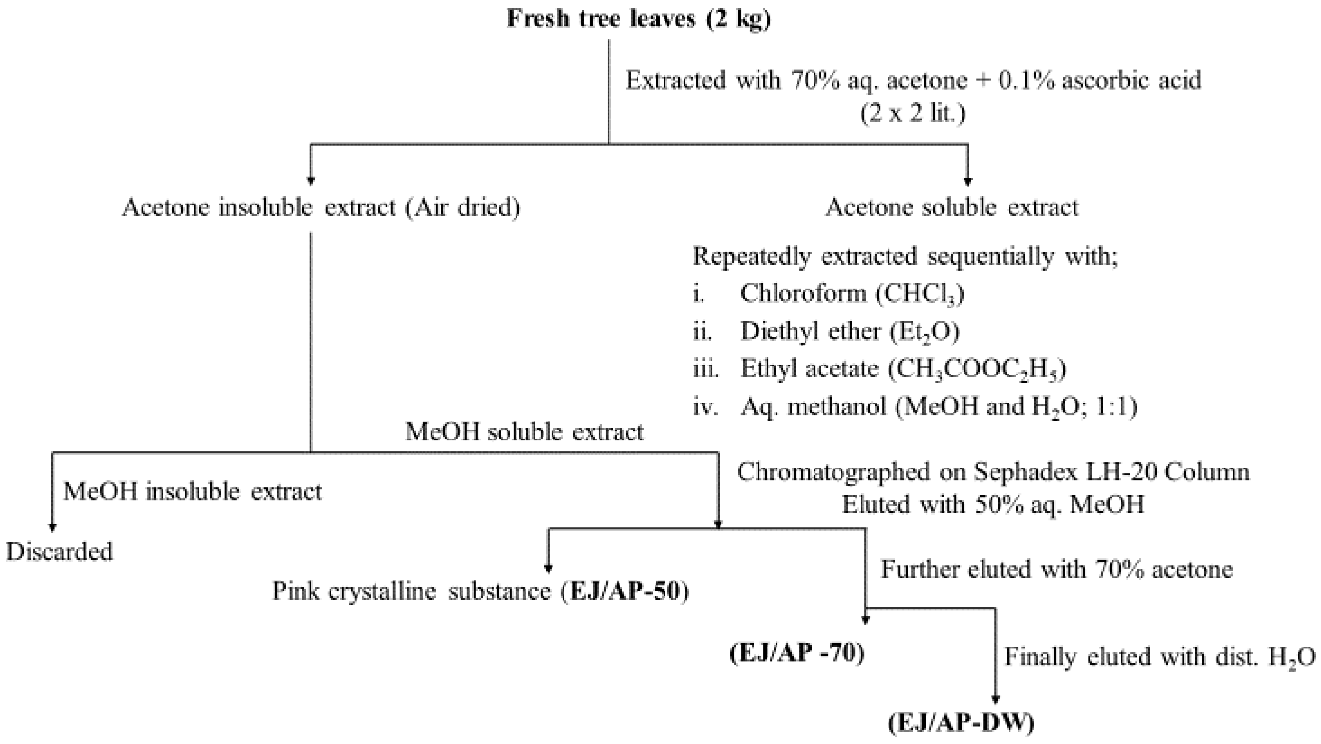

Figure S1. Schematic flowchart for extraction and isolation of proanthocyanidins from tree leaves

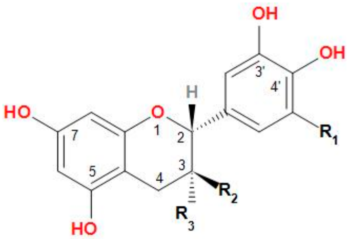

Flavan-3-ols

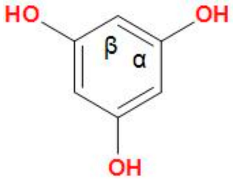

Phloroglucinol

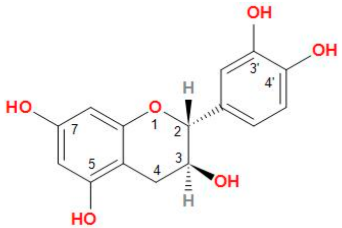

(+) Catechin
(2R, 3S)-*trans*-flavan-3-ols
(I)

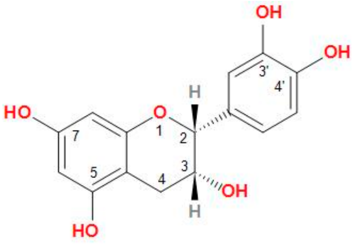

(-) Epicatechin
(2R, 3R)-*cis*-flavan-3-ols
(II)

**Flavonoid**	**R_1**	**R_2**	**R_3**
(+)-Catechin	H	OH	H
(−)-Epicatechin	H	H	OH
(+)-Gallocatechin	OH	OH	H
(−)-Epigallocatechin	OH	H	OH

Figure S2. Chemical structure of the compounds investigated in the study
