# Supplementary material for: Proanthocyanidins Modulate Rumen Enzyme Activities and Protein Utilization In Vitro
Source: Molecules. 2022 Sep 10;27(18):5870. doi: 10.3390/molecules27185870 (PMC9505871; doi:10.3390/molecules27185870)
Supplement: Supplementary file 1 [file molecules-27-05870-s001.zip › Supplementary table_PK.pdf]

Table S1. Degree of polymerisation and unit and compositions of sub-units of PAs in extracted plant fractions

**Fractions**	**Total Monomer**	**Total adduct**	**Degree of polymerization**	**Extension unit (%)**	**Terminal unit (%)**	**Sub-unit ratio**
EJ-50	0.684	5.338	7.80	89	11	Dp:Cy (66:34)
EJ-70	0.581	3.235	5.57	85	15	Dp:Cy (60:40)
EJ-DW	1.342	6.87	5.12	84	16	Dp:Cy:Pel (34:33:33)
AP-50	1.692	6.445	3.81	80	20	Dp:Cy: Pel: Mal (40:20:20:20)
AP-70	2.403	6.35	2.66	73	27	Dp:Cy (60:40)
AP-DW	0.672	4.201	6.25	84	16	Dp:Cy (60:40)

AP-50, AP-70 and AP-DW: Extract eluted with 50% of MeOH, 70% acetone and with distilled water respectively from *A. pendula*. EJ-50, EJ-70 and EJ-DW: Extract eluted with 50% of MeOH, 70% of acetone and with distilled water respectively from *E. jambolana*, Dp: delphinidin, Cy: cyanidin, Pel: pelargonidin and Mal: malvidin
